# Transcriptome Sequencing Reveals Molecular Characteristics of Spinal Cord Degeneration in MPTP-Induced Parkinson’s Disease Mice

**DOI:** 10.3390/biology15171503

**Published:** 2026-09-02

**Authors:** Linglong Xiao, Yaping Wu, Xinyuejia Huang, Hao Deng, Yang Wu, Wei Pan, Wei Wang, Mengqi Wang

**Affiliations:** 1 Department of Neurosurgery, The First Affiliated Hospital, Sun Yat-sen University, Guangzhou 510080, China; 2 Core Facility of West China Hospital, Sichuan University, Chengdu 610041, China; 3 Department of Neurosurgery, West China Hospital, Sichuan University, 37 Guoxue Alley, Chengdu 610041, China

**Keywords:** Parkinson’s disease, spinal cord degeneration, transcriptome, weighted gene co-expression network analysis

## Abstract

Parkinson’s disease (PD) involves not only the loss of dopaminergic neurons in the substantia nigra but also spinal cord degeneration. However, the molecular mechanisms of spinal cord degeneration remain unclear. This study investigated the spinal cord transcriptomic characteristics of MPTP-induced PD mice via transcriptome sequencing and weighted gene co-expression network analysis (WGCNA) to identify key gene modules and potential therapeutic targets. An MPTP-induced PD mouse model was established, and spinal cord transcriptome sequencing was conducted to screen differentially expressed genes (DEGs). Functional enrichment analysis, WGCNA for phenotype-correlated modules, and protein–protein interaction analysis were subsequently conducted to identify key genes. In total, 3473 DEGs were identified (1775 upregulated, 1698 downregulated). Downregulated genes were predominantly enriched in pathways related to oxidative phosphorylation and post-transcriptional regulation, whereas upregulated genes were associated with glutamatergic synapses, axonogenesis, and negative regulation of neurogenesis. Among the five co-expression modules, the brown and yellow modules were most strongly correlated with the PD phenotype, enriched in calcium signaling, inflammation, and spliceosome pathways. Key genes like Akt1, Nlrp3, Tgfb1, Lingo1, and Olig2 were upregulated, whereas Vps35 and Omg were downregulated. This study characterizes the spinal transcriptome of PD mice, suggesting that dysregulated post-transcriptional processes, abnormal oxidative phosphorylation, glutamatergic excitotoxicity, and neuroinflammation may be potential candidate mechanisms and vital involved factors in spinal cord degeneration. These findings provide novel insights into the pathological mechanisms of spinal cord degeneration in PD and lay a foundation for targeted therapy, deserving further investigation.

## Simple Summary

Parkinson’s disease (PD) is the second most common neurodegenerative disorder globally. Its pathological lesions are not restricted to dopaminergic neurons in the substantia nigra but also involve distal tissues such as the spinal cord. However, the molecular mechanisms of spinal cord degeneration remain unclear. This study used a mouse model of PD to investigate the genes that are upregulated or downregulated in the spinal cord. We identified over 3400 differentially expressed genes. Among these, some genes are responsible for regulating cellular energy production, while others are involved in synaptic transmission, axonal growth, and neuroinflammation. Through our analysis of the interactions among these genes, we identified that issues related to dysregulated post-transcriptional processes, abnormal oxidative phosphorylation, glutamatergic excitotoxicity, and neuroinflammation may be potential candidate mechanisms and vital involved factors in spinal cord degeneration of PD. Notably, several potential key genes emerged: some genes were found to be upregulated, indicating heightened neuroinflammation and disrupted neuronal repair, whereas others were downregulated, suggesting impaired energy balance and a loss of the protective myelin sheath surrounding axons. This work broadens our understanding of PD beyond the brain, and provides new insight for developing treatments to alleviate spinal cord degeneration and improve patients’ quality of life.

## 1. Introduction

Parkinson’s disease (PD) is the second most prevalent neurodegenerative disease globally, affecting approximately 1% of individuals aged 60 years and older [1]. The primary pathological hallmark in PD is the degeneration of dopaminergic neurons in the substantia nigra (SN), resulting in motor dysfunction [2]. Previous studies have demonstrated that early pathological changes in PD initially occur in the nigrostriatal system, and progressively spread to other brain regions, including the cortex and hippocampus as the disease progresses [3,4].

In recent years, investigations have revealed that, in addition to cerebral pathology, certain clinical manifestations in PD patients are associated with spinal cord involvement. These manifestations encompass both motor symptoms, including gait disturbances and non-motor symptoms such as constipation and urinary or sexual dysfunction [5,6]. Numerous clinical and experimental studies have demonstrated the presence of lesions in the spinal cord in PD. Lesions were detected in the dorsal horn of the spinal cord in all 6 patients with PD examined by the Braak group [7]. Neuronal loss in the intermediolateral nucleus at the T2 and T9 spinal segments of PD patients was reported by Wakabayashi, Takahashi, and colleagues [8,9,10]. In a large-sample spinal cord pathology study conducted by Beach et al., Lewy bodies were detected in the gray matter of 16 out of 17 PD patients [11]. These findings indicate that degeneration occurs in the spinal cord of PD patients, but the mechanism is poorly understood. Previous studies have investigated the mechanisms of spinal cord degeneration using the 1-methyl-4-phenyl-1,2,3,6-tetrahydropyridine (MPTP)-induced PD model. As early as 1985, Lyden et al. [12] found that ^3^H-MPTP could accumulate in the spinal cord, and altered glucose metabolism was observed in the spinal cord [13]. Furthermore, Samantaray et al. [14,15] observed neurodegeneration in cervical and lumbar spinal cord neurons in an MPTP-induced PD model and confirmed the presence of an MPTP-metabolizing system in the spinal cord. They also reported a 30% loss of motor neurons in the spinal cord of both MPTP-induced PD models and PD patients [15,16,17], and this mechanism was closely related to neuroinflammation [5]. Meanwhile, Zhang et al. [18] observed significantly increased microglial reactivity in the spinal cord of MPTP-induced mouse PD models, with activated microglia implicated in local neuronal death [16,19,20,21]. Moreover, pathologically upregulated calpain activity in the spinal cord may lead to motor dysfunction through neuronal death mediated by inflammatory responses [16]. These findings further strengthen the association between neuroinflammation and spinal cord degeneration in PD. The above evidence indicates that spinal cord degeneration may represent one of the important contributors to the various symptoms observed in PD. However, most researchers have focused their therapeutic investigations primarily on brain pathology, with relatively limited attention paid to spinal cord degeneration. This may partially account for the suboptimal therapeutic efficacy of PD over the years. Nevertheless, existing studies on spinal cord degeneration in PD are insufficient to fully characterize the mechanisms underlying spinal cord degeneration in PD, necessitating further comprehensive investigation. Weighted Gene Co-expression Network Analysis (WGCNA) is a systems biology approach that delineates gene association patterns across various samples [22]. By analyzing the correlations between gene sets and phenotypes, this method can be employed to identify highly coordinated gene modules and determine potential candidate biomarker genes or therapeutic targets [23]. This method holds significant value for elucidating the characteristics and mechanisms of MPTP-induced spinal cord degeneration in PD. However, no study has yet applied this approach to the investigation of spinal cord degeneration in PD.

The primary objective of this study is to investigate the molecular characteristics within spinal cord tissues of PD mice. We employed transcriptome sequencing analysis of spinal cord tissue from MPTP-induced mouse PD models and applied WGCNA to screen for gene modules highly correlated with phenotypes. Meanwhile, by comparing the transcriptomic sequencing results of spinal cord tissue between the healthy control group and the MPTP-induced PD mouse model group, this study aims to provide a more comprehensive understanding of the dysregulated genomic landscape associated with spinal cord degeneration. This approach will facilitate the identification of therapeutic target genes with clinical potential, thereby offering novel perspectives and strategies for the treatment of PD and establishing a foundation for subsequent mechanistic investigations into PD-associated spinal cord degeneration.

## 2. Materials and Methods

### 2.1. Experimental Animals and Study Design

**Figure 1:**
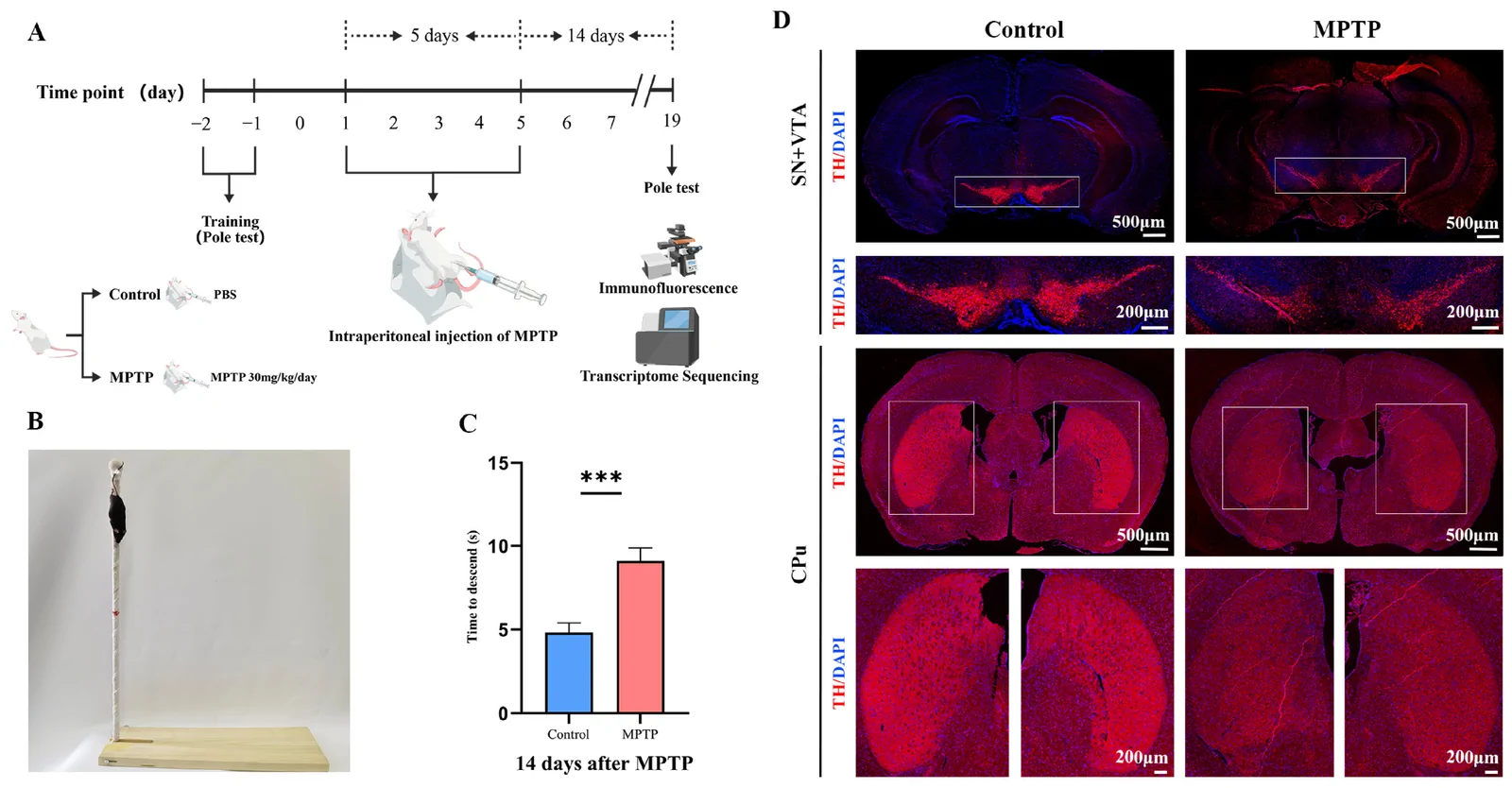
Workflow of the research and behavioral assessments (Created with BioGDP.com [30]). (**A**) Study design and experimental workflow. (**B**) Representative image of the mouse pole test. (**C**) Statistical analysis of the pole test results. *n* = 6 per group, *** *p* < 0.001. (**D**) TH immunofluorescence staining results in the SN, VTA, and CPu regions.

Male C57BL/6J mice, weighing 20–25 g (8–10 weeks old), were provided by the Laboratory Animal Center of the First Affiliated Hospital of Sun Yat-sen University. All mice were housed in a specific pathogen-free (SPF) environment with controlled light, temperature, and humidity (12-h light/dark cycle, constant temperature of approximately 25 °C, and relative humidity of approximately 55%). Throughout the experimental period, all mice had ad libitum access to standard chow and water. The mice were randomly and equally divided into two groups: the control group and the MPTP group (Figure 1A). All experiments were approved by the Clinical Research and Laboratory Animal Ethics Committee of the First Affiliated Hospital of Sun Yat-sen University.

### 2.2. Subacute MPTP-Induced Mouse Model of PD

The PD model was established according to the methods described in previous studies [24,25,26,27]. For the MPTP group, 90 mg MPTP powder (Sigma-Aldrich, Darmstadt, Germany) was dissolved in 30 mL sterile PBS. Mice (*n* = 6) received daily intraperitoneal (i.p.) injections of MPTP at a dose of 30 mg/kg for 5 consecutive days, whereas control mice (*n* = 6) were administered an equal volume of sterile PBS intraperitoneally.

### 2.3. Behavioral Assessment

The pole test was performed on the experimental mice according to the methods described by Zhang et al. and Huang et al. [27,28]. A vertical pole measuring 50 cm in length and 1 cm in diameter was used, with a wooden ball attached to its top. The wooden ball was wrapped in gauze. A mouse was placed on top of the wooden ball and allowed to climb down the pole to the base (Figure 1B). The time taken for the mouse to reach the base of the pole was recorded. All experimental animals were subjected to two days of behavioral training. The test commenced 14 days after the last injection of MPTP. Each mouse underwent three separate tests with a 1-h inter-test interval, and the average time was calculated for subsequent statistical analysis.

### 2.4. Spinal Cord Tissue Preparation

Mice were anesthetized via intraperitoneal injection of 1.25% tribromoethanol, followed by transcardial perfusion with phosphate-buffered saline (PBS). Immediately after perfusion, the vertebral column was rapidly dissected, and the cervical enlargement segment of the spinal cord was carefully extracted. The isolated spinal cord tissue was then promptly snap-frozen in liquid nitrogen and stored for subsequent transcriptomic sequencing.

### 2.5. Immunofluorescence Staining

Brain samples were carefully isolated for histological analysis. Briefly, under deep anesthesia, mice were transcardially perfused with PBS followed by 4% paraformaldehyde (PFA). Brain samples were post-fixed in 4% PFA at 4 °C overnight and cryoprotected in 30% sucrose solution before being snap-frozen on dry ice. Samples were sectioned into 20 μm slices using a Leica CM1950 cryostat (Leica Biosystems Nussloch GmbH, Wetzlar, Germany). Cryosections were incubated in blocking buffer containing 1× PBS, 5% bovine serum albumin (BSA) (Cat#: SW3015, Solarbio, Beijing, China), and 0.3% Triton™ X-100 (Cat#: T8787, Sigma-Aldrich) at room temperature for 1 h, and then incubated with the primary antibody [anti-TH (1:1000, Cat#: ab6211, Abcam, Cambridge, UK)] at 4 °C overnight. Sections were rinsed three times in 1× PBS before incubation with the fluorochrome-conjugated secondary antibody, Alexa Fluor^®^ 568 Donkey anti-Rabbit IgG (1:200, Cat#: ab175470, Abcam, Cambridge, UK), at room temperature for 1 h. After being rinsed three times, sections were counterstained with DAPI-containing mounting medium to visualize cell nuclei and imaged using a confocal laser scanning microscope.

### 2.6. Screening of Differentially Expressed Genes

Differentially expressed genes (DEGs) between the MPTP group (*n* = 6) and the control group (*n* = 6) were identified from RNA-seq data using the DESeq2. Genes exhibiting significant changes were selected based on a *p*-value < 0.05 and log_2_|fold change| > 0.

### 2.7. Construction of a Weighted Gene Co-Expression Network

The co-expression network of DEGs was constructed using the R package “WGCNA” [29]. The network construction process comprised the following major steps: (1) selection of an appropriate soft-thresholding power (weighting coefficient) and definition of the similarity matrix; (2) transformation of the similarity matrix into an adjacency matrix using a power adjacency function; (3) conversion of the adjacency matrix into a topological overlap matrix (TOM); (4) generation of a hierarchical clustering tree based on the TOM-based dissimilarity (dissTOM) using hierarchical clustering; and (5) identification of modules as branches of the hierarchical clustering tree using the dynamic tree cut method.

### 2.8. Identification of Biologically Significant Modules

The primary objective of Weighted Gene Co-Expression Network Analysis (WGCNA) is to associate key genes from co-expression networks with external clinical traits. The correlation between gene modules and clinical features was analyzed as follows: (1) The module eigengene (ME) was calculated for each module, with the ME representing the overall expression level of that module. (2) The module membership (MM) was identified, where MM indicates the correlation between the gene expression profile and the ME. A higher absolute value of MM signifies greater importance of the gene within the module. (3) The gene significance (GS) and module significance (MS) were extracted. The MS is defined as the average of the absolute GS values for all genes within a given module. A higher absolute GS value indicates greater biological significance of the gene [29].

### 2.9. Functional Enrichment Analysis

To gain further insights into the functions of the DEGs within the modules most significantly associated with spinal cord tissue in the MPTP group, enrichment analysis was performed. *p* < 0.05 was set as the cutoff, and the results were visualized using R software (version 4.5.0). Gene set enrichment analysis (GSEA) was conducted using the R package “clusterProfiler” to identify enriched pathways, with an adjusted *p*-value < 0.05 set as the cutoff criterion.

### 2.10. Featured Gene Selection and PPI Network Construction

The candidate featured genes were uploaded to the STRING database, and the protein–protein interactions (PPIs) among these genes were retrieved using the online tool, based on which a network diagram was constructed. Cluster analysis was performed to extract tightly connected regions within the biological network, isolate significant modules, and hide disconnected nodes in the network, thereby generating the corresponding PPI network diagram. The FPKM values of differentially expressed genes were analyzed, and Welch’s *t*-test was employed to assess statistical differences between the MPTP group and the control group.

### 2.11. Statistical Analysis

For the DEGs analysis of RNA-seq data, we performed the analysis using DESeq2. Genes exhibiting significant changes were selected based on a *p*-value < 0.05 and log_2_|fold change| > 0. The data other than RNA-seq are presented as means ± standard deviations and were analyzed using SPSS 26.0 software. Statistical analysis was conducted using two-tailed Student’s *t*-tests or Welch’s *t*-test, when the data were normally distributed. A Mann–Whitney U test was performed, when the data were not normally distributed. A *p*-value < 0.05 was considered to indicate statistically significant differences.

## 3. Results

### 3.1. Successful Establishment of a Mouse PD Model via MPTP Administration

To simulate the manifestations of clinical PD, MPTP was administered to experimental mice to induce PD-like lesions. Behavioral assessments such as the pole test were conducted to detect motor dysfunction triggered by MPTP. The results showed that mice in the MPTP group spent significantly more time climbing from the apex to the base of the vertical pole compared with control mice (Figure 1C). Furthermore, to further validate the reliability of the model, we recapitulated the core pathological hallmarks of PD using immunofluorescence staining. As shown in the representative images (Figure 1D), TH-positive dopaminergic neurons were visibly decreased in the substantia nigra (SN) and ventral tegmental area (VTA), and TH fluorescence intensity was reduced in the caudate putamen (CPu) of MPTP-treated mice. These changes represent the core pathological features of PD. Collectively, these results verified that a stable and reliable mouse PD model was successfully established using MPTP.

### 3.2. DEGs in the Spinal Cord Between the MPTP and the Control Group

**Figure 2:**
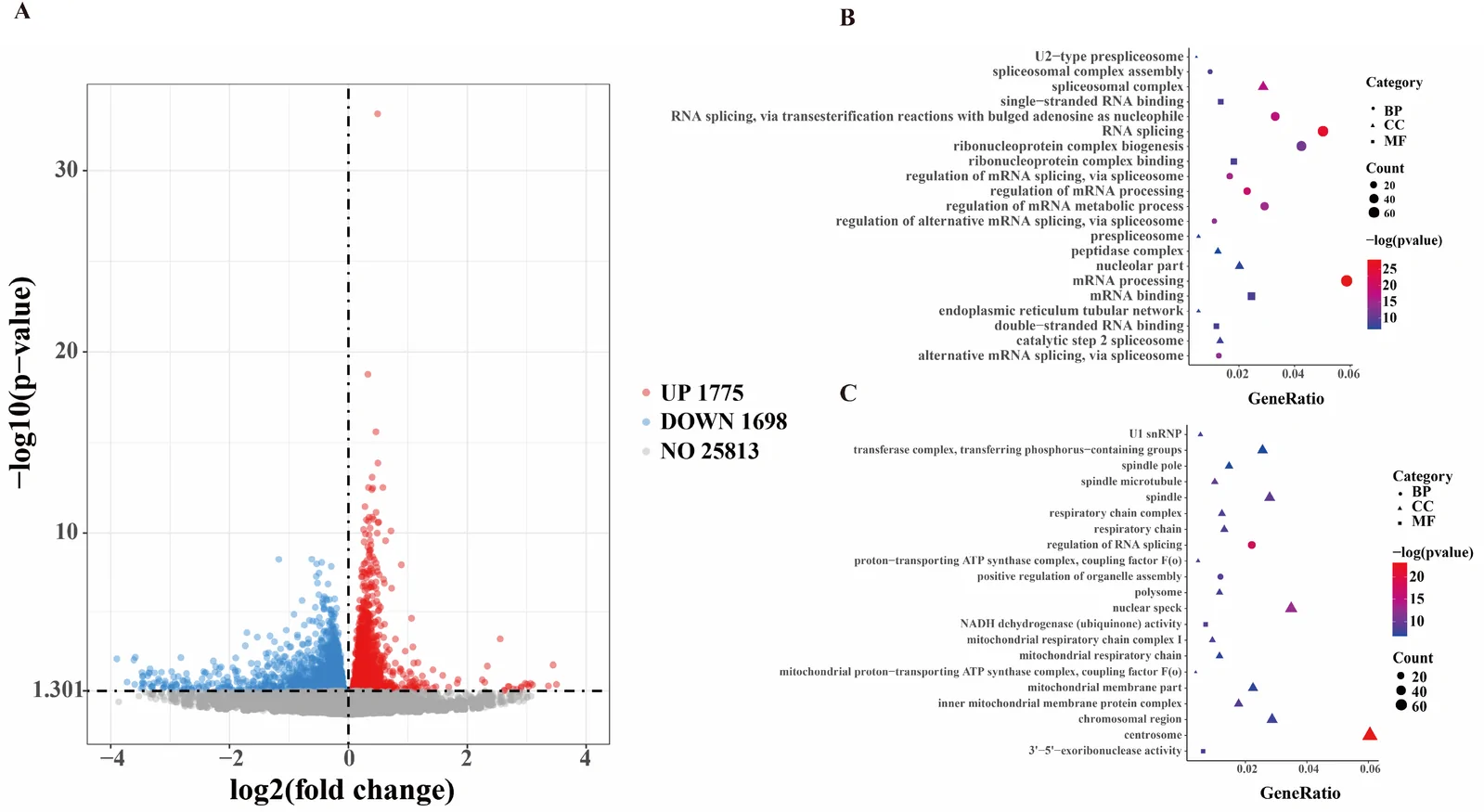
Volcano plot and enrichment analysis of differentially expressed genes (DEGs). (**A**) Volcano plot of DEGs. Red, upregulated genes in the spinal cord tissues of the MPTP group; blue, downregulated genes in the spinal cord tissues of the MPTP group. (**B**,**C**) Enriched Gene Ontology (GO) terms for downregulated DEGs. *n* = 6 per group.

In this study, spinal cord tissues from the MPTP-induced PD model and the control group were analyzed, and a total of 3473 DEGs were identified between the two groups. Among these, 1775 DEGs were found to be upregulated in the spinal cord tissues of PD mice, whereas 1698 DEGs were downregulated (Figure 2A).

### 3.3. Molecular Pathways Associated with Spinal Cord Tissues in MPTP-Induced PD Models

**Figure 3:**
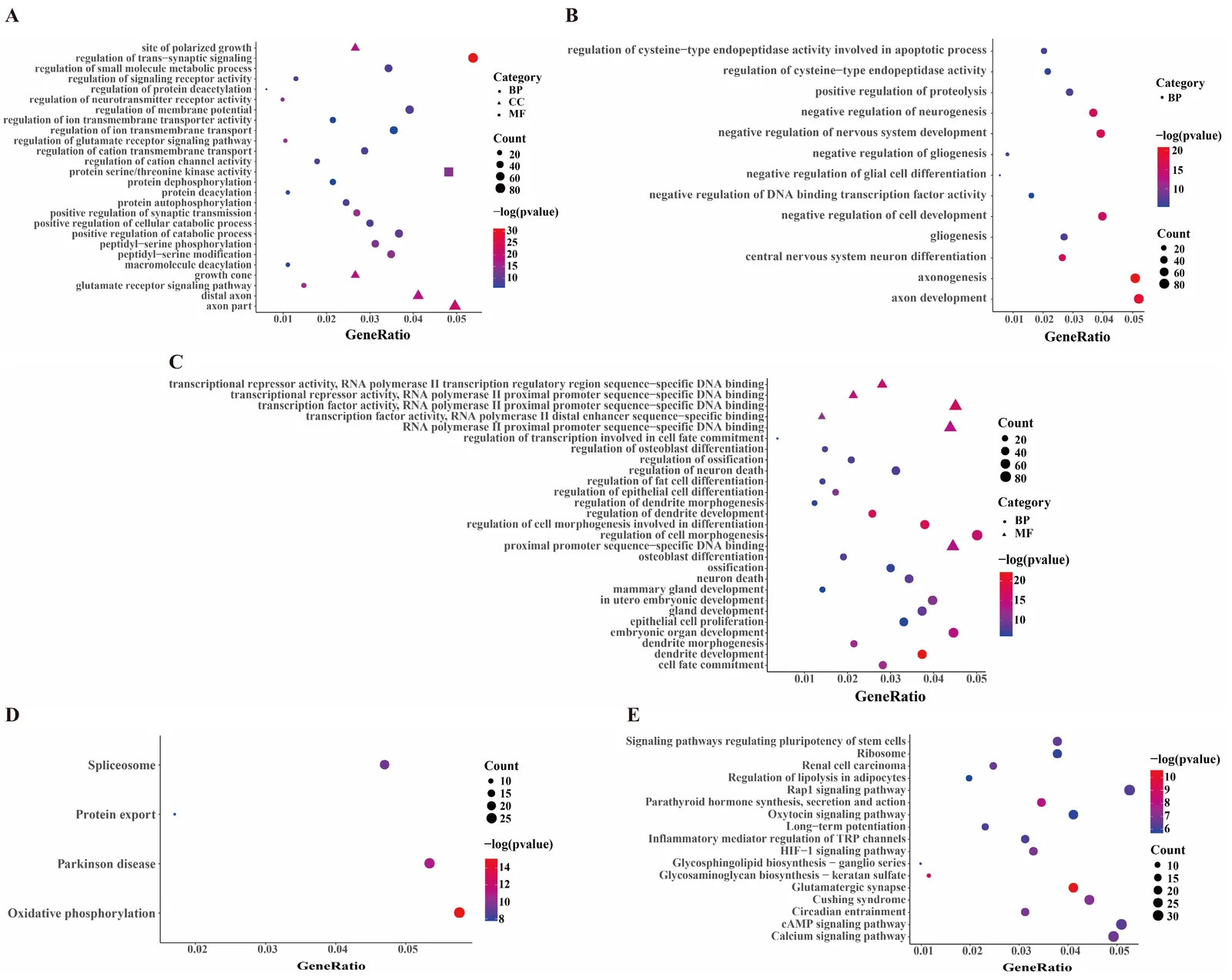
(**A**–**C**) Enriched GO terms for upregulated DEGs. (**D**) Enriched pathways for downregulated DEGs. (**E**) Enriched pathways for upregulated DEGs. *n* = 6 per group.

To explore the potential biological functions of these DEGs, functional enrichment analysis was performed. The results revealed that both upregulated and downregulated DEGs in the spinal cord tissues of the PD group were significantly enriched in diverse biological processes (BP). For instance, downregulated DEGs were enriched in BP including RNA splicing and mRNA processing (Figure 2B). Upregulated DEGs were enriched in regulation of trans-synaptic signaling, negative regulation of neurogenesis, negative regulation of nervous system development, negative regulation of cell development, axonogenesis, axon development, and dendrite development (Figure 3A–C). For cellular component (CC), downregulated DEGs exhibited prominent enrichment in the centrosome (Figure 2C), whereas upregulated DEGs were markedly enriched in the axon part and distal axon (Figure 3A). Regarding molecular function (MF), upregulated DEGs were enriched in transcription factor activity and RNA polymerase II proximal promoter sequence-specific DNA binding (Figure 3C), whereas no significantly enriched MF terms were detected among downregulated DEGs. Subsequently, pathway analysis was conducted on the selected DEGs to gain further insights. Downregulated DEGs were enriched in pathways such as oxidative phosphorylation (Figure 3D), and upregulated DEGs were enriched in pathways such as glutamatergic synapse (Figure 3E).

### 3.4. Gene Enrichment Results in Spinal Cord Tissue of MPTP-PD Mice

**Figure 4:**
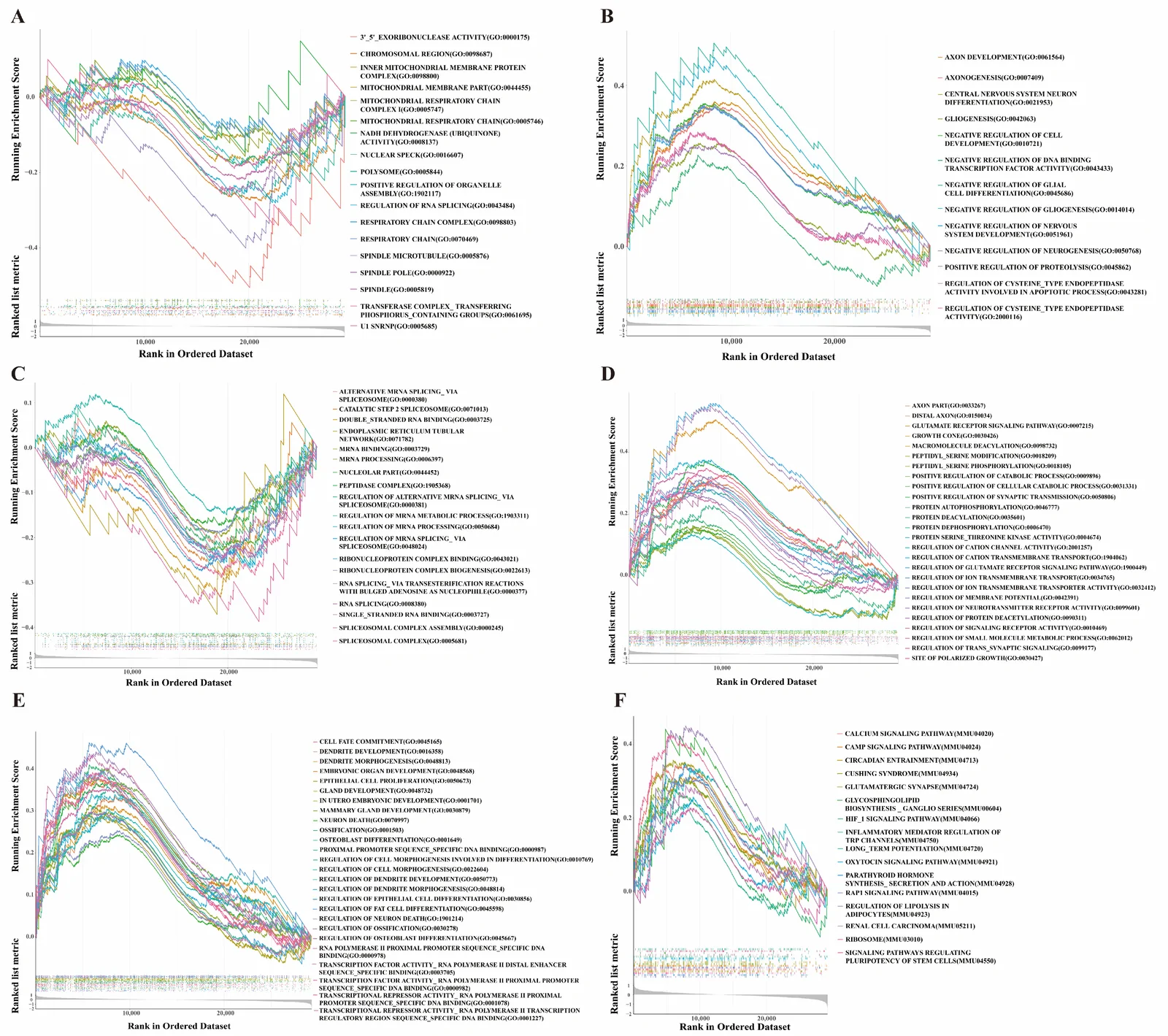
Gene set enrichment analysis (GSEA). (**A**–**F**) Enriched gene sets in the spinal cord tissues of the MPTP group. *n* = 6 per group.

Significant differences were observed in the DEGs between the spinal cord tissues of the PD group and the control group, suggesting that the pathological changes in the spinal cord involve multiple distinct molecular mechanisms. To investigate the molecular functions and signaling pathways associated with these DEGs, GSEA was performed to compare relevant pathways between the MPTP and control groups. The results showed that, compared with the control group, the PD group exhibited enrichment in pathways related to the mitochondrial respiratory chain (Figure 4A), negative regulation of glial cell differentiation (Figure 4B), negative regulation of gliogenesis (Figure 4B), regulation of mRNA metabolic processes (Figure 4C), signaling pathways associated with glutamate receptor regulation (Figure 4D), pathways involved in the regulation of neuronal death (Figure 4E), and glutamatergic synapse-related signaling pathways (Figure 4F). These findings indicated that, in the MPTP-induced PD model, multiple pathogenic pathophysiological processes occur in the spinal cord tissue, which may be associated with the symptoms of PD.

### 3.5. Weighted Gene Co-Expression Modules of MPTP-PD Spinal Cord Tissues

**Figure 5:**
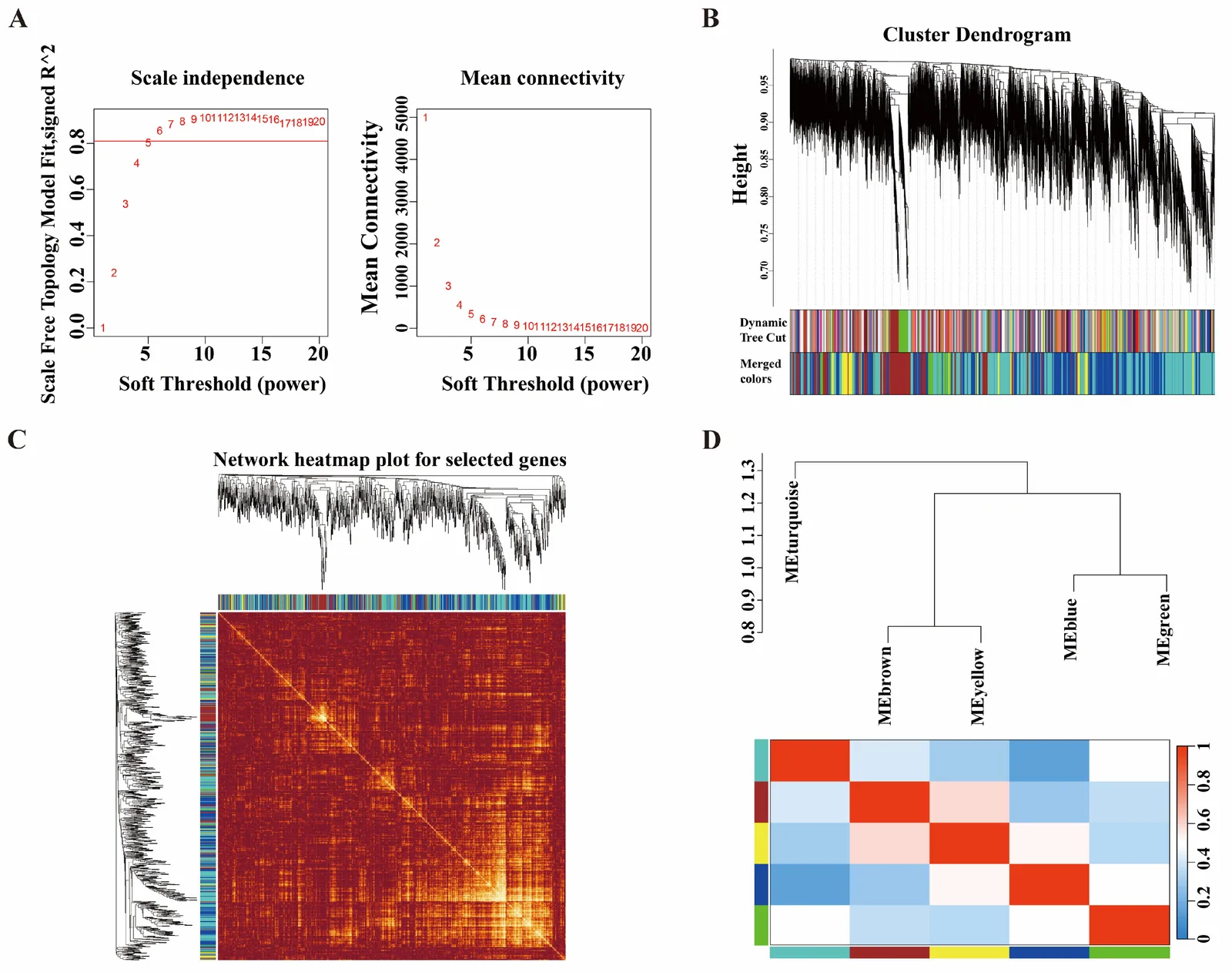
Construction of co-expression modules. (**A**) Determination of the soft-threshold power. (**B**) Cluster dendrogram of genes. Genes that failed to cluster into any of the defined modules were assigned to the gray module. Every gene represents a line in the hierarchical cluster. (**C**) Network heatmap plot of genes selected for WGCNA network construction. (**D**) Heatmap plot of the adjacencies in the eigengene network. *n* = 6 per group.

WGCNA was employed to construct a gene co-expression network. Connectivity between genes in the network formed the scale-free network distribution when 0.8 was set as the correlation coefficient threshold, and the soft-thresholding power was selected as 5 (Figure 5A). Subsequently, hierarchical clustering of all genes was performed, and co-expression modules were identified using the dynamic tree-cutting method. A total of 5 distinct co-expression modules were ultimately constructed (Figure 5B). Among these, the turquoise module contained the greatest number of genes, followed by the blue module. Genes that failed to be assigned to any specific module were classified into the grey module, which collected genes with no obvious co-expression patterns. The module–gene clustering heatmap revealed that the identified modules were well-separated (Figure 5C). The adjacency heatmap of module eigengenes further illustrated the interrelationships among distinct modules (Figure 5D): one cluster consisted of the turquoise and blue modules, while the other comprised the brown, yellow, and green modules. Notably, module pairs within the same cluster (such as the brown–yellow module pair) exhibited stronger adjacency relationships, suggesting coordinated expression patterns between them.

### 3.6. Gene Co-Expression Modules Associated with Characteristics of Spinal Cord Lesion in Mice

**Figure 6:**
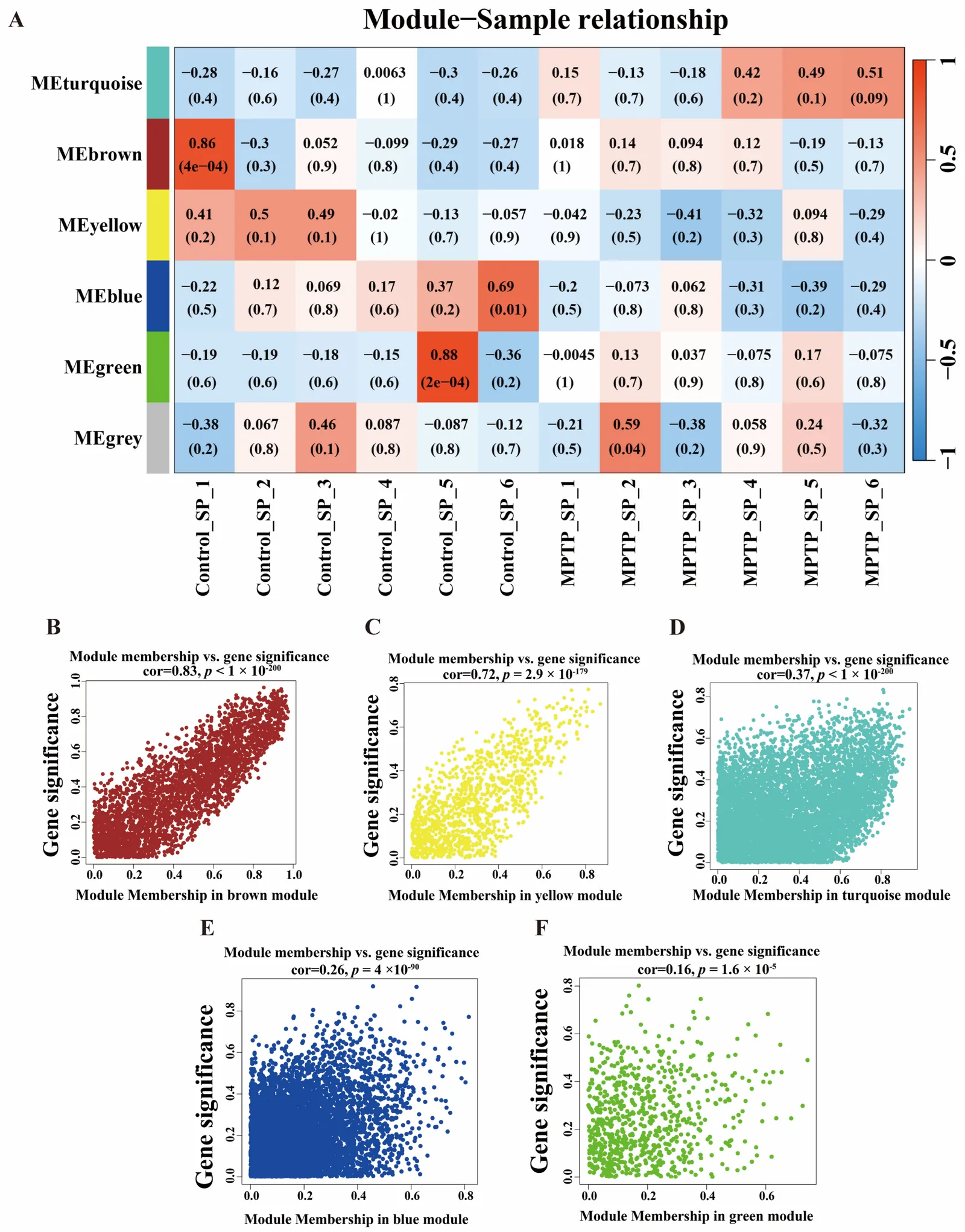
Relationship between modules and sample. (**A**) Heatmap of the module–sample relationships. (**B**–**F**) Scatterplots of gene significance (GS) vs. module significance (MS) in modules. *n* = 6 per group.

To investigate the biological significance of the identified modules, we analyzed the correlations between the module eigengenes and the sample phenotypes. Module–sample association analysis revealed that several modules were significantly correlated with distinct experimental treatment conditions (Figure 6A). Among these, the brown module exhibited a strong positive correlation with the control group, whereas the turquoise module was significantly associated with the MPTP group. These findings suggest that the genes within these modules may play critical roles in the corresponding biological processes. To further investigate the reliability of these phenotype-associated modules, we examined the correlation between Gene Significance (GS) and Module Membership (MM) for the genes within each module. The results showed that the yellow and brown modules displayed the highest correlation coefficients between GS and MM (cor = 0.72 and 0.83, respectively; *p* = 2.9 × 10^−179^ and *p* < 1 × 10^−200^; Figure 6B,C), indicating that genes with high MM were also strongly associated with the experimental phenotypes. This finding suggested that the yellow and brown modules are the most representative phenotype-related modules.

### 3.7. Functional Enrichment Analysis Results of Interesting Gene Sets

**Figure 7:**
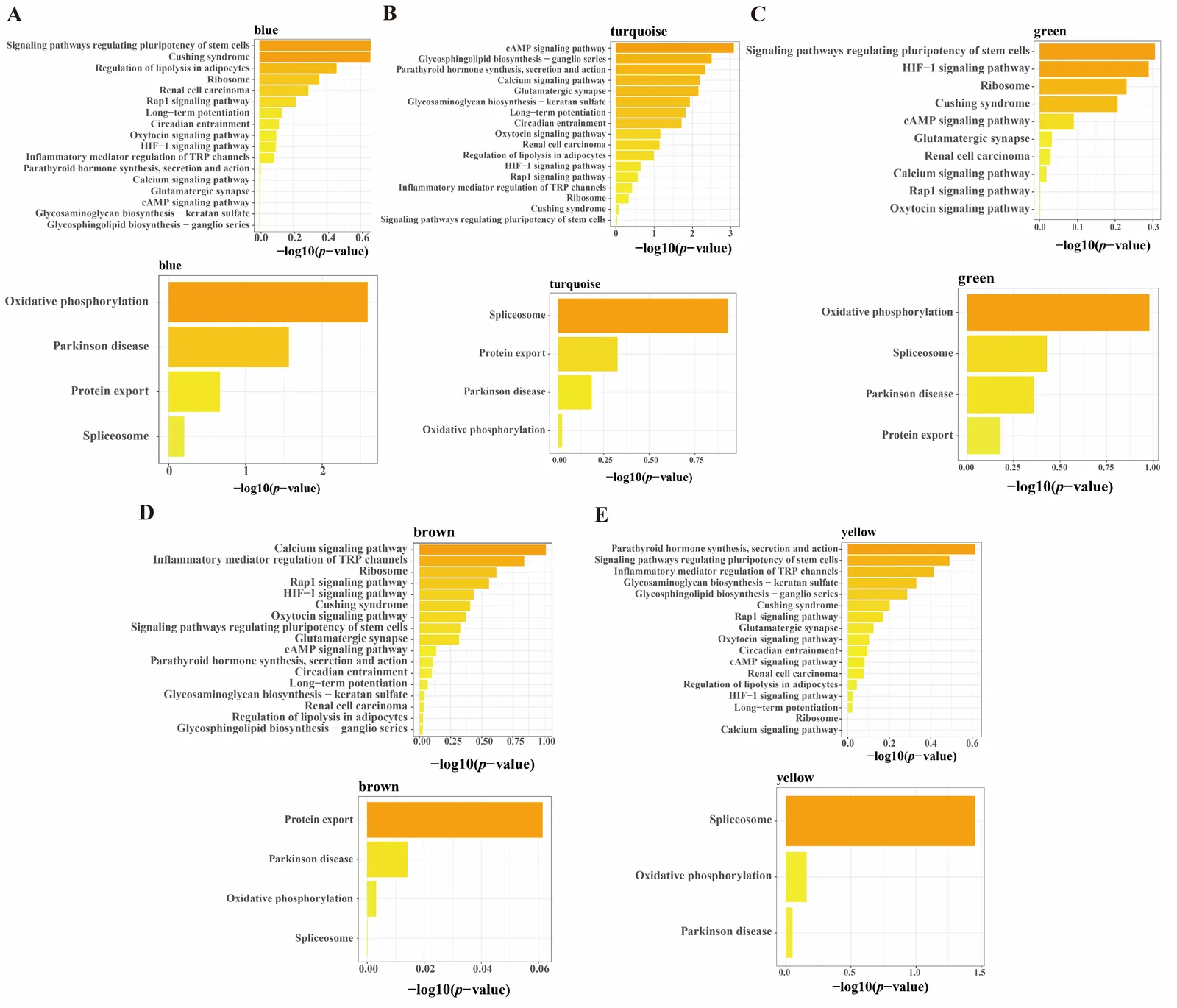
Gene ontology analysis and Kyoto Encyclopedia of Genes and Genomes (KEGG) pathway enrichment results for genes in modules related to the spinal cord of the MPTP group. (**A**) The enrichment results for genes in the blue module. (**B**) The enrichment results for genes in the turquoise module. (**C**) The enrichment results for genes in the green module. (**D**) The enrichment results for genes in the brown module. (**E**) The enrichment results for genes in the yellow module. *n* = 6 per group.

To elucidate the biological functions of the key modules identified via WGCNA, functional enrichment analyses were performed on the genes contained within each module. KEGG pathway enrichment analysis of the blue module revealed that its genes were primarily enriched in pathways related to the regulation of stem cell pluripotency and oxidative phosphorylation. Furthermore, disease enrichment analysis indicated that the genes in this module were closely associated with neurodegenerative diseases, such as PD (Figure 7A). Genes in the turquoise module were predominantly enriched in the cAMP signaling pathway, glycosphingolipid biosynthesis—ganglio series pathway, calcium signaling pathway, and glutamatergic synapse pathway (Figure 7B). For the green module, significant enrichment was observed in signaling pathways regulating pluripotency of stem cells, the HIF-1 signaling pathway, and oxidative phosphorylation-related pathways (Figure 7C). Genes in the brown module were significantly enriched in the calcium signaling pathway and pathways related to the inflammatory mediator regulation of TRP channels (Figure 7D). The yellow module exhibited enrichment in pathways such as the spliceosome (Figure 7E). These findings indicated that the key modules identified in this study are primarily involved in biological functions related to oxidative phosphorylation, glutamatergic synapses, inflammatory regulation, calcium signaling, the spliceosome, and neurodegenerative diseases.

### 3.8. Construction of PPI Network and Analysis of Potential Hub Genes

**Figure 8:**
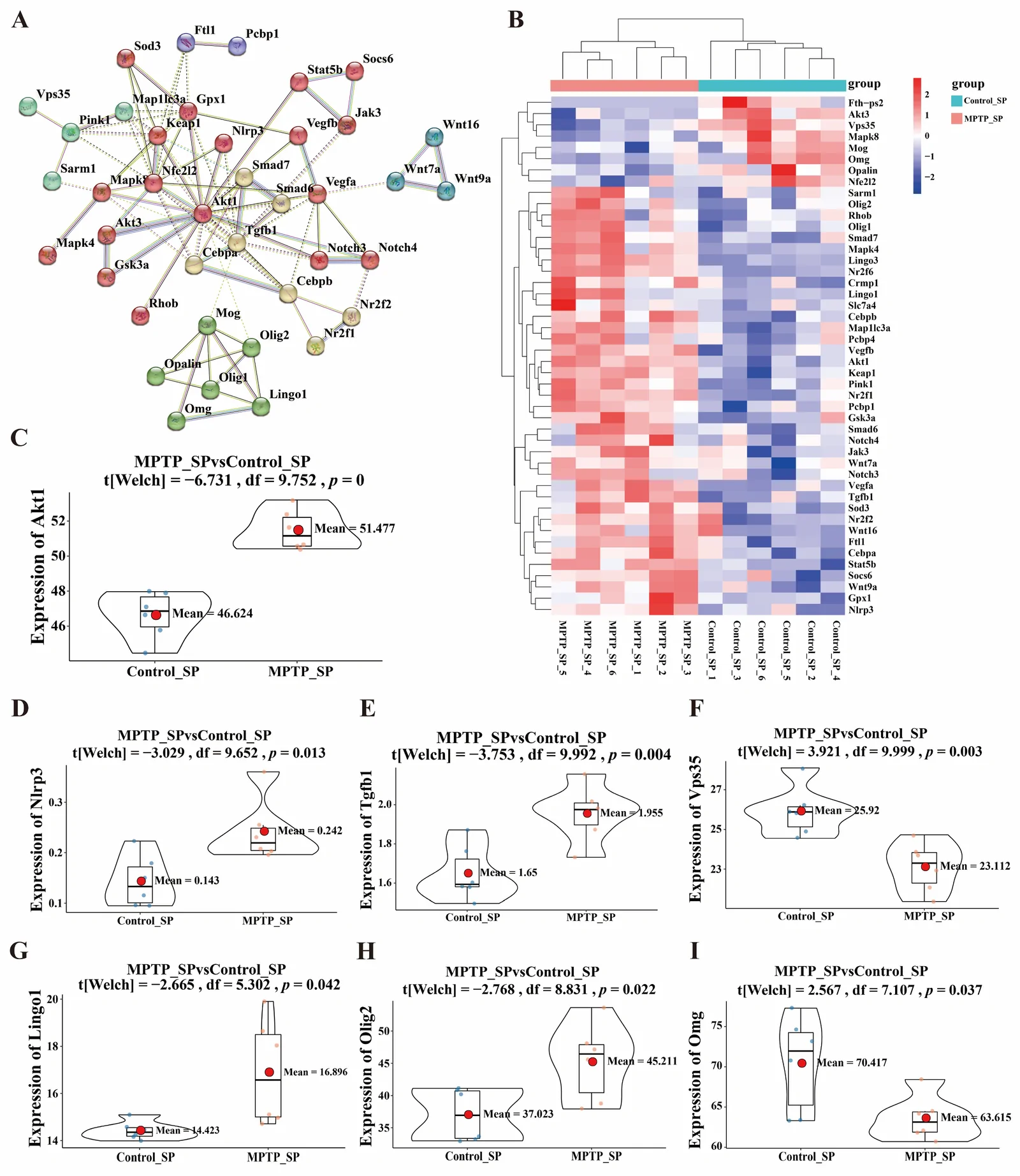
Identification of key genes. (**A**) The protein–protein interaction (PPI) network. (**B**) Heatmap of the candidate key genes. (**C**–**I**) The violin plots of potential key genes in the MPTP spinal cord. *n* = 6 per group.

To further clarify the intrinsic interactions among DEGs, the STRING database was employed to establish a PPI network. Genes such as Akt1, NLRP3, and Tgfb1 were identified as key nodes within the PPI network (Figure 8A), implying that these genes may exert vital biological functions in the spinal cord of PD mice. Additionally, white matter-related genes such as Olig2, Lingo1, and Omg exhibited tight correlations with the above key genes. As visualized in the heatmap (Figure 8B), the candidate key genes could clearly discriminate the MPTP group from the control group. To investigate the expression levels of these potential key genes, the FPKM values of the key genes were analyzed. The results showed that the expression levels of Akt1, Nlrp3, Tgfb1, Lingo1, and Olig2 were significantly upregulated in the MPTP group relative to the control group, whereas Vps35 and Omg were markedly downregulated (Figure 8C–I).

## 4. Discussion

PD is the second most common neurodegenerative disorder worldwide. Researchers have long focused their studies on the core pathological regions within the brain, while spinal cord involvement and its molecular mechanisms have received insufficient attention. In this study, DEGs in the spinal cord tissues of PD mice were identified via transcriptome sequencing. A weighted gene co-expression network was subsequently constructed to identify key gene modules associated with PD, and their related biological functions and pathways were analyzed. Our findings provide a new perspective for deciphering the pathological basis of spinal cord degeneration in PD.

First, behavioral assessments were carried out on the MPTP-induced PD mouse model. Pole test results revealed significant motor dysfunction in the MPTP group. Meanwhile, immunofluorescence staining results indicated substantial loss of dopaminergic neurons in the SN and VTA region, further validating the stability and reliability of the established PD model. These findings were consistent with those of previous studies employing MPTP-induced PD models [25,27,31,32]. This provides a reliable experimental foundation for transcriptomic sequencing and analysis.

Subsequently, transcriptomic sequencing and differential analysis in this study revealed large-scale aberrant alterations in the spinal cord tissues of PD mice. Among these, RNA splicing and mRNA processing are encompassed within the domain of post-transcriptional regulation. Studies have shown that classical post-transcriptional regulatory mechanisms, such as abnormal m6A modification, are involved in the progression of PD. For instance, Teng et al. [33] elucidated the molecular mechanism by which methyltransferase METTL14-mediated m6A modification maintains endoplasmic reticulum homeostasis and calcium balance in dopaminergic neurons through the regulation of Atp2a3 gene expression, thereby protecting neuronal survival. Although our results did not show abnormal m6A modification, they identified the downregulation of RNA splicing and mRNA processing, suggesting that post-transcriptional regulatory processes are involved not only in brain pathology but may also influence spinal cord pathology in PD. In addition, we observed a decrease in the expression of the centrosome, a crucial organelle involved in cell division, which may be one of the factors responsible for the disruption of spinal cord axonal remodeling. However, no studies have yet investigated its role in the spinal cord in PD. Oxidative phosphorylation is a central process in mitochondrial energy metabolism, and mitochondrial dysfunction has been established as a hallmark pathological change in neurodegenerative diseases [34]. In this study, downregulation of oxidative phosphorylation-related signaling was observed, suggesting that spinal cord degeneration in PD may be associated with impaired energy metabolism, which is consistent with previous findings. Ali et al. [35] proposed that abrogation of electron transport complex I during oxidative phosphorylation leads to reduced ATP levels in dopaminergic neurons, subsequently inducing apoptosis and thereby exacerbating the progression of PD. Moreover, altered glucose metabolism in the spinal cord was also detected by Schwartzman et al. [36] in MPTP-induced PD monkey models. These results suggest that cellular energy metabolism in the spinal cord is abnormal under PD conditions. In conjunction with aforementioned verified mechanisms and the transcriptomic abnormalities identified in this study, these findings suggest that this abnormality may be closely associated with pathological changes and related behavioral deficits, but the precise causal relationship and pathogenic mechanisms remain to be further investigated.

We also observed that genes associated with axonal and dendritic development were markedly upregulated in the spinal cord of PD mice, which contrasts with previously reported white matter fiber and axonal degeneration in the spinal cord of PD mice. This seemingly paradoxical phenomenon can be interpreted as follows: aberrant remodeling of axons and dendrites may serve as a compensatory reaction against dopaminergic neuron loss, or alternatively, represent an early pathological hallmark of neurodegeneration. GSEA further verified significant enrichment of gene sets associated with axonogenesis and axon development in the spinal cord of PD mice, indicating concurrent axonal injury and reparative processes. Nevertheless, such axonal plasticity is presumably limited. As the disease progresses and compensatory mechanisms become exhausted, axonal injury eventually outweighs axonal repair, ultimately leading to motor dysfunction. Previous studies have demonstrated that the spinal cord contains abundant glutamatergic projections, and their abnormal activation can lead to excessive glutamate release, thereby mediating neuronal excitotoxicity and subsequently triggering calcium influx, neuroinflammation, and mitochondrial dysfunction [37,38]. These pathological processes may aggravate spinal neuronal and axonal injury, collectively contributing to the progression of spinal cord degeneration in PD. Although numerous studies have demonstrated that excitotoxicity caused by excessive glutamate release is an important mechanism in several neurodegenerative diseases including PD [37,39,40], studies on its role and mechanisms in spinal cord degeneration in PD remain limited. This study identified significant enrichment of the glutamatergic synapse pathway in the spinal cord of PD mice, suggesting that glutamatergic synapse-related pathways may represent a direction for future exploration. In summary, the above findings indicate that multiple pathological pathways, including mitochondrial dysfunction, dysregulation of RNA transcription and post-transcriptional regulation, axonal injury, and abnormal synaptic transmission, may serve as important involved factors to spinal cord degenerative changes in PD, which is worthy of further in-depth research in the future.

Additionally, through WGCNA, the brown and yellow modules were identified as the most phenotype-correlated modules. Genes in the brown module were enriched in the calcium signaling pathway and the regulation of TRP channels by inflammatory mediators. This finding is consistent with previous reports of abnormal upregulation of calpain [16,41,42,43]. And this further suggests that neuroinflammation and calcium homeostasis imbalance may serve as important involved factors to spinal cord degeneration in PD. Meanwhile, the yellow module was enriched in the spliceosome pathway. Abnormalities in the spliceosome can lead to defects in mRNA processing, thereby affecting the expression of proteins associated with neuronal and axonal survival. This observation aligns with the enrichment of downregulated DEGs in RNA splicing and mRNA processing, further suggesting that post-transcriptional dysregulation may constitute a vital link in PD-related spinal cord degeneration.

Furthermore, NLRP3 is a critical driver of neuroinflammation that can exacerbate neuronal and axonal injury. This study found that its expression is upregulated in the spinal cord in PD, which is generally consistent with the results of previous studies. Studies have indicated that the MPTP-induced PD model is characterized by microglial activation and neuroinflammation in the spinal cord, and that microglial activation and neuroinflammation are associated with local neuronal death [16,18,19,20,21]. However, it has not been demonstrated whether spinal cord neuroinflammation in PD is mediated by the NLRP3. Overall, NLRP3-mediated neuroinflammation is likely a key trigger of spinal cord pathology in PD, and this requires further investigation to confirm. Given that neuroinflammation serves as a critical driver of motor neuron loss and axonal degeneration in the spinal cord, NLRP3-mediated spinal cord neuroinflammation may represent a promising direction for future pharmacological research and therapeutic development. Akt1 is a core molecule in cell survival signaling pathways. In this study, Akt1 expression was upregulated, which contradicts previous findings. Earlier studies reported markedly reduced phosphorylation of Akt family proteins in SN dopaminergic neurons of PD [44,45]. Furthermore, Salinas et al. [46] demonstrated that Akt1 overexpression could alleviate reactive oxygen species (ROS)-mediated apoptosis and exert neuroprotective effects. These previous studies support the notion that aberrant Akt1 signaling participates in the regulation of cell survival in neurodegenerative disorders such as PD. Similarly, Tgfb1 is an anti-inflammatory cytokine, and the results of this study are inconsistent with previous findings. Karampetsou et al. [47] proposed that the Tgfb1 signaling pathway plays a crucial role in the differentiation, maintenance, and synaptic function of dopaminergic neurons, and that Tgfb1 treatment exhibits a neuroprotective effect on dopaminergic neurons in animal models of PD. However, in this study, Tgfb1 expression was upregulated. The inconsistency between the expression of Akt1 and Tgfb1 observed in this study and that reported previously may be attributable to the detection time point, which was still within the early stage of the disease. At this stage, the spinal cord may still be in a compensatory phase, and the upregulation of Akt1 and Tgfb1 expression may represent a compensatory protective response. As the disease progresses, persistent chronic inflammation may abrogate these endogenous protective mechanisms, ultimately leading to neuronal and axonal loss. Although both Akt1 and Tgfb1 were upregulated at this experimental stage, their dynamic changes at different time points following PD model establishment, including both short-term and long-term periods, remain to be further investigated in the future.

We also observed changes in markers associated with white matter injury. Lingo1 serves as a negative regulator of oligodendrocyte differentiation and myelination; its upregulation may inhibit myelin repair and exacerbate white matter injury. Olig2 participates in the differentiation of oligodendrocyte progenitor cells, and its aberrant expression may disrupt remyelination. In contrast, Omg is a myelin-associated protein, and its downregulation may reflect structural disruption of the myelin sheath. These results are consistent with the spinal cord axonal degeneration reported in previous studies using the MPTP-induced PD model [5]. Combined with direct evidence of spinal cord degeneration in PD from previous studies and the spinal cord transcriptomic expression changes in the present study, these findings indicate that under PD conditions, the spinal cord undergoes not only gray matter neuronal loss but also degeneration of white matter tracts formed by axonal bundles. This type of damage may be closely associated with gait disturbances and other axial symptoms presented in patients with PD, which requires validation in future studies.

Furthermore, we observed downregulated expression of vacuolar protein sorting 35 (Vps35) in the spinal cord of PD mice. Vps35 is a core component of the retromer complex involved in regulating protein trafficking and retrieval. Mutations in VPS35 are closely associated with the pathophysiology of PD. Accumulating evidence demonstrates that the D620N mutation in Vps35 is one of the causative factors for late-onset autosomal dominant familial PD and also contributes to sporadic PD [48,49]. This mutation induces mitochondrial dysfunction and enhanced neuroinflammation, and is associated with α-synuclein aggregation [49,50]. Clinically, patients harboring this mutation typically present with slowly progressive parkinsonism [51]. At the molecular level, the Vps35 D620N mutation has been shown to perturb mitochondrial membrane potential and aggravate mitochondrial dysfunction [52]. However, the aforementioned studies on Vps35 have focused exclusively on the brain, without investigation of the spinal cord. In this study, Vps35 expression was found to be downregulated in the spinal cord, suggesting that spinal cord neurons may similarly face an increased risk of protein homeostasis imbalance due to aberrant Vps35 expression.

Several clinical studies have investigated spinal cord tissue from PD patients. By examining the spinal cords of deceased PD patients, these studies found evidence of neuronal loss and detected Lewy bodies and α-synuclein in the spinal cord [7,8,9,10,11,53], indicating that the spinal cord in human PD exhibits the same degenerative changes as those in the brain. Unfortunately, however, these clinical studies did not investigate the mechanism in depth. There are still no studies investigating the mechanisms of spinal cord degeneration in human PD. In the future, methods such as transcriptomic sequencing could be used to validate the findings of this study in human PD spinal cord tissue, thereby laying the foundation for clinical translation.

Although this study investigated the transcriptomic characteristics of the spinal cord in PD mice, several limitations remain. First, the MPTP-induced PD model cannot fully simulate the chronic progressive nature of human PD. In the future, transcriptome sequencing of postmortem specimens from PD patients should still be conducted under appropriate conditions to explore abnormal changes in the spinal cord in human PD, which will provide an important foundation for clinical translation. Second, the findings are based on transcriptomic data analysis, and the potential key genes have not been validated by qRT-PCR, Western blot or proteomics and immunohistochemistry, and alterations in protein expression levels and their associated biological functions require further validation through molecular biology experiments. Third, this study was limited to tissue-level analysis of the spinal cord, the specific gene expression profiles of distinct cell types, such as neurons, microglia, and oligodendrocytes, await further elucidation via single-cell RNA sequencing approaches. Finally, the causal relationships between the identified key genes, key signaling pathways, and spinal cord degeneration remain unclear, and their specific regulatory mechanisms need to be elucidated through in vivo and in vitro gene interference experiments.

## 5. Conclusions

Taken together, this study revealed the transcriptomic characteristics of spinal cord degeneration in PD mice through transcriptomic sequencing and WGCNA. Notably, dysregulated post-transcriptional processes, abnormal oxidative phosphorylation, glutamatergic excitotoxicity, and neuroinflammation may be potential candidate mechanisms and vital involved factors to spinal cord degeneration, deserving further investigation. These transcriptomic expression changes provide a theoretical basis and potential therapeutic targets for developing novel treatment strategies targeting symptoms associated with spinal cord degeneration in PD. They also provide new insights into understanding non-substantia nigra lesions in PD. Future research should focus on functional validation and mechanistic exploration of key genes involved in spinal cord degeneration, thereby providing experimental evidence for the development of targeted therapeutic strategies.

## Data Availability

The RNA-seq raw data in this article have been deposited in the Genome Sequence Archive in National Genomics Data Center, China National Center for Bioinformation (GSA: CRA045591) that are publicly accessible at https://ngdc.cncb.ac.cn/gsa/, accessed on 29 June 2026. Other data will be made available upon reasonable request.
